# Cigarette smoke aggravates asthma via altering airways inflammation phenotypes and remodelling

**DOI:** 10.1111/crj.13718

**Published:** 2023-11-14

**Authors:** Qiong Huang, Yan Li, Chenduo Li, Xin Zhang, Xiaonan Du, Yan Chen, Chris J. Corrigan, Wei Wang, Sun Ying

**Affiliations:** ^1^ Department of Prenatal Diagnostic Center, Guangzhou Women and Children's Medical Center Guangzhou Medical University Guangzhou China; ^2^ Department of Immunology, School of Basic Medical Sciences Capital Medical University Beijing China; ^3^ Department of Otorhinolaryngology Head and Neck Surgery, Beijing Tongren Hospital, Capital Medical University, Beijing Institute of Otorhinolaryngology, Key Laboratory of Otorhinolaryngology Head and Neck Surgery, Ministry of Education Beijing Key Laboratory of Nasal Diseases Beijing China; ^4^ Faculty of Life Sciences & Medicine, School of Immunology & Microbial Sciences, Asthma UK Centre in Allergic Mechanisms of Asthma King's College London London UK

**Keywords:** airways hyperresponsiveness, airways inflammation, airways remodelling, asthma, cigarette smoke

## Abstract

**Introduction:**

Many asthmatic patients are exposed to cigarette smoke actively or passively, which contributes to asthma exacerbation and poor control. This study is to explore the effects of cigarette smoke on pathological changes in murine surrogate of asthma.

**Methods:**

C57BL/6 mice were sensitised and challenged with ovalbumin (OVA) to establish a surrogate of asthma and then administered with cigarette smoke extract (CSE). Airways hyperresponsiveness (AHR) was measured using the Flexivent system. Histological staining (haematoxylin‐eosin [HE], periodic acid Schiff [PAS], Congo red and Masson's trichrome) was employed to measure pathological changes in sections of lung tissue of experimental mice. Enzyme‐linked immunosorbent assay (ELISA) was used to measure the concentrations of total and OVA‐specific IgE, cytokines and chemokines (eotaxin‐1, IL‐13, IL‐1β, TNF‐α, IL‐17A, IL‐33) in the lung tissue homogenates. Immunoreactivity for vWF and α‐SMA in lung tissue sections was detected by immunohistochemistry.

**Results:**

Exposure of the animals to CSE significantly reduced OVA‐induced AHR, the number of eosinophils in bronchoalveolar lavage fluid (BALF) and eosinophils infiltrating into the lung tissue, as well as concentrations of some cytokines in lung homogenate. In contrast, it significantly enhanced the number of macrophages and M2 in BALF, as well as collagen deposition, smooth muscle thickness and alveolar destruction in lung tissue.

**Conclusion:**

CSE inhibits OVA‐induced AHR, changes inflammation ‘phenotypes’, while accelerates some aspects of airways remodelling, which might contribute to worse symptoms and be refractory to anti‐inflammation therapies for asthmatics.

## Graphical abstract

Cigarette smoke inhibited allergen‐induced airways hyperrespinsiveness, changed inflammation ‘phenotypes’, while accelerated airways remodelling.

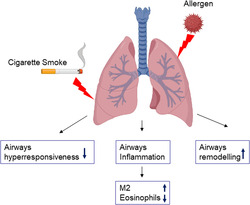

## 1 INTRODUCTION

Asthma is a common, chronic respiratory disease associated with substantial morbidity and mortality. An estimated 241 million people suffer from asthma worldwide.^1^ According to the WHO, there were 383 000 deaths attributed to asthma in 2015.

Asthma is a heterogeneous disease in terms of the spectrum and severity of clinical symptoms as well pathological changes in the airways, which include nonspecific airways hyperresponsiveness (AHR) to bronchoconstricting stimuli, inflammation and remodelling. The cells and cytokines causing airways inflammation in asthma vary between individuals, giving rise to the concept of ‘phenotypes’ of inflammation. While the precise aetiology of these phenotypes is unknown, their description is commonly crystallised into ‘Type‐2‐high’, observed in approximately 50% of adult asthmatics, and the remainder, or ‘Type‐2‐low’. The former is characterised by a prominent eosinophilic infiltrate accompanied by the overexpression of type 2 cytokines, including IL‐4, IL‐5 and IL‐13, overexpressed by Th2 and ILC2 following stimulation with epithelial alarmin cytokines including IL‐33, TSLP and IL‐25. ‘Type‐2‐low’ airways inflammation is more heterogeneous and its aetiology correspondingly less clear but may reflect the effects of cytokines released from Th1, Th17 or ILC3. Airways remodelling may involve a wide variety of structural changes in the epithelium and subepithelium, including goblet cell hyperplasia, neoangiogenesis, laydown of collagens and other interstitial proteins and smooth muscle hypertrophy. The aetiology of these changes is not clearly delineated, but the fact that they may appear early in children who suffer from asthma suggests that they are not always an aftermath of chronic airways inflammation.^2^, ^3^

Chronic obstructive pulmonary disease (COPD) caused by exposure to smoke, typically tobacco smoke, causes progressive, irreversible airways obstruction and, in some patients, additional alveolar destruction (emphysema).^4^ Cigarette smoke exposure, whether from active smoking, maternal smoking or second‐ or third‐hand exposure is a well‐documented trigger for asthma exacerbation and poor control.^1^, ^5^ International consensus documented, for example, in the 2023 Global Initiative for Asthma (GINA) Report suggests that some patients may be diagnosed with ‘asthma‐COPD overlap’ (ACO, a simple descriptor for patients who have characteristics of both asthma and COPD). Discriminating asthma from COPD may be challenging, especially in smokers and older adults, since both diseases may be associated with a degree of irreversible airways obstruction. Lung function testing is critical to confirm AHR, the key pathognomonic feature of asthma. In the present study, we address the hypothesis that cigarette smoke aggravates allergen‐induced pathological changes relevant to disease severity and refractoriness to therapy in this murine surrogate.

## 2 MATERIALS AND METHODS

### 2.1 Experimental animals

Male C57BL/6 mice (8–10 weeks) were obtained from Vital River Laboratories (Beijing, China) and housed in a pathogen‐free environment in the Department of Laboratory Animal Sciences, Capital Medical University, Beijing, China.

### 2.2 Preparation of soluble CSE

Ten cigarettes (Marlboro) (tar 11 mg, nicotine 0.8 mg, CO 11 mg) were lit successively, and the smoke sucked through 5 mL phosphate‐buffered saline (PBS) using a micro‐negative pump uniformly to dissolve it.^6^ The solution was then filtered through a 0.22 μm sterile filter. This concentration of cigarette smoke extract (CSE) was arbitrarily regarded as 100%. CSE was used immediately once prepared, avoiding repetitive freeze‐thawing.

### 2.3 Administration of CSE, ovalbumin (OVA) and PBS control

**FIGURE 1:**
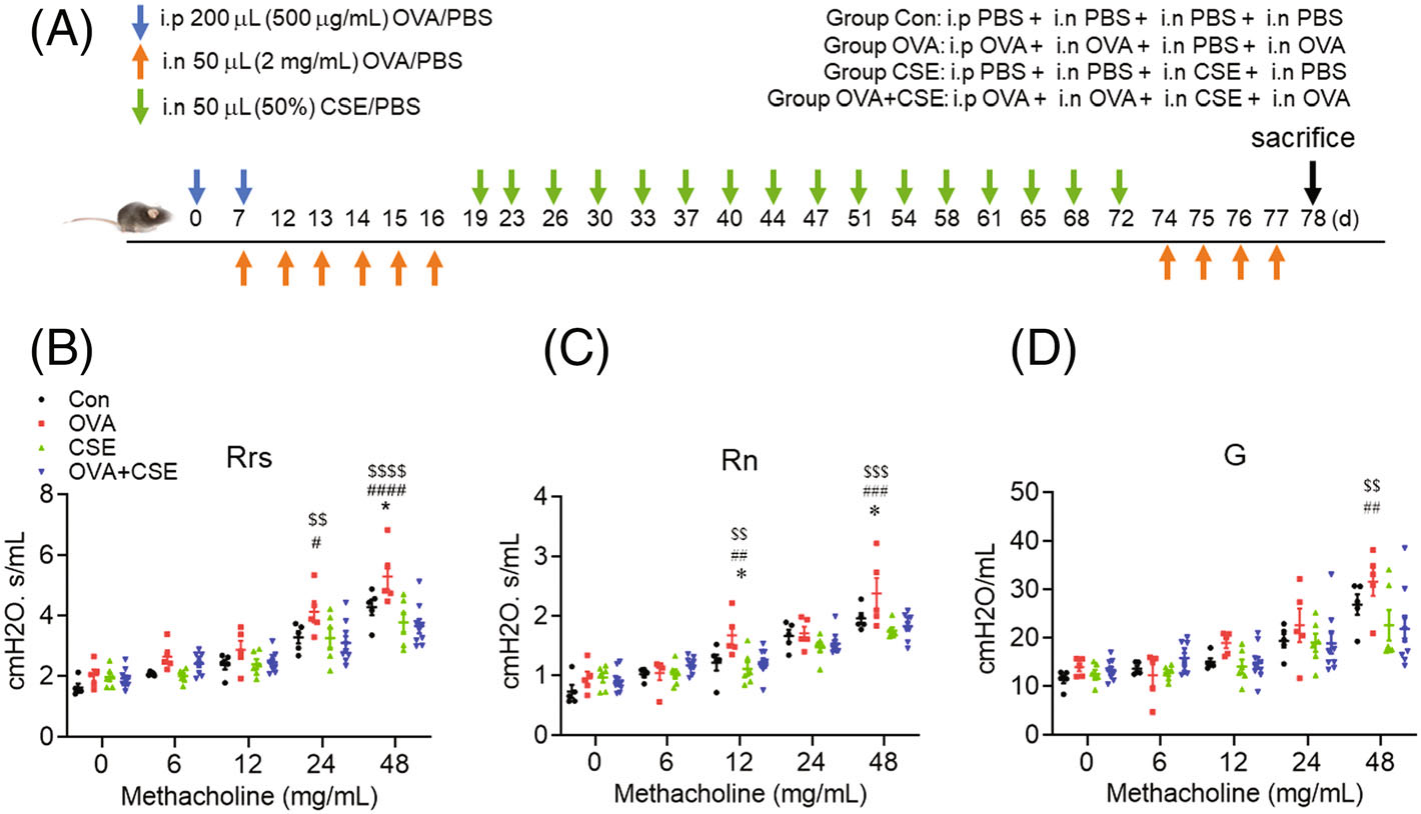
The effect of CSE on OVA‐induced AHR. (A) Schedule of challenges in the murine model. (B−D) Changes in lung function parameters in response to methacholine. *p < 0.05 (OVA group vs. Con group). ^#^ *p* < 0.05; ^##^ *p* < 0.01; ^###^ *p* < 0.001; ^####^ *p* < 0.0001 (OVA group vs. CSE group). ^$$^ *p* < 0.01; ^$$$^ *p* < 0.001; ^$$$$^ *p* < 0.0001 (OVA group vs. OVA+CSE group). Data are expressed as the mean ± SEM. CSE, cigarette smoke extract; G, tissue damping; OVA, ovalbumin; Rn, central airway resistance; Rrs, total resistance.

The murine surrogate of asthma was established by challenging OVA‐sensitised (100 μg/200 μL in PBS plus alum) animals per‐nasally with OVA (100 μg/50 μL in PBS) intermittently over a 16 day period as previously described.^7^ Subsequently, the ‘asthmatic’ mice were challenged with CSE (50%/50 μL in PBS) per‐nasally twice weekly for 8 weeks and then rechallenged with OVA (100 μg/50 μL in PBS) per‐nasally once daily for 4 consecutive days (Figure 1A). Individual animals were randomly assigned to four groups treated with PBS alone (always exposed to diluent control), OVA alone (exposed to OVA followed by diluent control and then exposed to OVA again), CSE alone (exposed to diluent control before the CSE exposure and then exposed to diluent control again) and both OVA and CSE (exposed to OVA followed by CSE and then exposed to OVA again).

### 2.4 Measurement of lung function

Animals were anaesthetised by intraperitoneal injection of 2% sodium pentobarbital at a dosage of 80 mg/kg 24 h after the final OVA challenge and then intubated endotracheally. Airways responsiveness to nebulised methacholine was measured using the FlexiVent system (SCIREQ, Canada).^8^ Readouts included Rrs, Rn and G, reflecting overall resistance of the airways, resistance of the central airways and tissue resistance, respectively.

### 2.5 Collection and analysis of BALF

Bronchoalveolar lavage fluid (BALF) was collected from the anaesthetised, intubated animals by injecting and withdrawing PBS (0.8 mL) twice through the tracheal intubation needle.^8^ Briefly, after centrifugation at 1200 rpm for 10 min at 4°C, the cell pellet was resuspended in 0.5 mL PBS. Cytospins were prepared and fixed with 4% paraformaldehyde for cellular differential counts. Congo red staining was used to identify eosinophils and other cell types in BALF. A total of 300 cells was evaluated on each slide under high magnification for statistical analysis.

### 2.6 Immunofluorescence

Immunofluorescence was used to explore the change of alternatively activated macrophages (M2) (CD68^+^CD206^+^) in cytospins prepared from BALF, using commercial primary rabbit antibody against mannose receptor (CD206) (ab64693, Abcam) and mouse antibody against CD68 (ab31630, Abcam) as well as secondary antibodies Alexa Fluor® 594‐goat anti‐rabbit (ZF‐0516, ZSGB‐BIO) and Alexa Fluor®488‐goat anti‐mouse (S8801, GuanXingyu). Signals were examined using fluorescence microscopy (Y‐TV55, Nikon, Japan).

### 2.7 Histochemical staining

After tying off the root of right main bronchus with a ligature, the right lungs of the experimental mice were removed for further use, and then the left lung was inflated via the tracheal intubation needle by mild infusion of 4% paraformaldehyde at a persistent fluid pressure of 25 cm for 5 min.^9^ After the root of left main bronchus tied off with a ligature, the left lung of experimental mice was removed and fixed in 10% formalin for 24 h, then dehydrated, paraffin‐embedded and sectioned (4 μm).^8^ Histological staining with haematoxylin‐eosin (HE), periodic acid Schiff (PAS), Congo red and Masson's trichrome was employed to assess inflammatory cellular infiltration, mean linear intercept (MLI), mucus secretion, eosinophil infiltration and collagen deposition, respectively.

HE staining score was used to quantify global infiltration of inflammatory cells around the airways and blood vessels in the lung sections, that is, 0 (no cells), 1 (a few cells), 2 (one layer of cells), and 3 (2–4 layers of cells) and 4 (more than 4 layers of cells).^10^ The MLI technique was used to assess alveolar integrity, which equalled to linear length divided by linear intercept alveolar number.^10^ The PAS score was used to quantify the percentages of the areas of the airways expressing mucin, that is, 0 (≤5%), 1 (5%–25%), 2 (25%–50%), 3 (50%–75%) and 4 (≥75%).^11^ Eosinophil infiltration around the airways and blood vessels in the sections was quantified as percentages of the numbers of Congo red‐staining cells in the total nucleated cells.^8^ Collagen deposition in the lung sections was expressed as the percentages of the areas stained with aniline blue in the field.^10^ For all analyses, at least 15 fields were assessed in tissue sections from each animal (avoiding large blood vessels and airways) using an optical microscope (Y‐TV55, Nikon, Japan) and Image‐Pro Plus 6.0 for image and statistical analysis.

### 2.8 Immunohistochemical staining

Commercial antibodies against vWF (von Willebrand factor) (AB7356, Millipore) and α‐SMA (alpha‐smooth muscle actin) (ab32575, Abcam), used to detect immunoreactivity of vWF (endothelial cells) and α‐SMA (airway smooth muscle) for quantifying blood vessels and airway smooth muscle, respectively, as well as AffiniPure Mouse Anti‐Rabbit (117 304, Jackson Immuno Research) and Peroxidase‐conjugated AffiniPure Donkey Anti‐Mouse antibody (116 722, Jackson Immuno Research) were used for procedures as previously described.^10^ The numbers of vWF positive stained blood vessels and smooth muscle thickness around the airways and vessels in the sections were measured using Image‐Pro Plus 6.0.

### 2.9 Measurements of the concentrations of serum IgE and cytokines in lung tissue

After weighing, the right lungs of the mice were homogenised in the presence of protease inhibitor (04693159001, Roche) and then stored at −80°C until analysed. Commercial ELISA kits were used to measure the concentrations of total and OVA‐specific IgE (88‐50460, Invitrogen) in the serum, as well the concentrations of eotaxin‐1 (BMS6008TEN, Invitrogen), IL‐13 (88‐7137, Invitrogen), IL‐1β (88‐7013, Invitrogen), IL‐6 (88‐7064, Invitrogen), IL‐17A (88‐7371, Invitrogen) and IL‐33 (88‐7333, Invitrogen) in the lung homogenates according to the manufacturers' instructions.

### 2.10 Statistical analysis

All data are presented as the mean±SEM. Data management and analysis were performed using GraphPad Prism 8.0 (La Jolla, US). Differences between experimental groups were analysed using the two‐tailed unpaired *t*‐test for parametric statistical evaluation or the two‐tailed Mann–Whitney test for nonparametric statistical evaluation. The lung function measurement data were analysed using two‐way ANOVA. For all tests, *p*‐values less than 0.05 were considered significant.

## 3 RESULTS

### 3.1 The effect of CSE on OVA‐induced AHR

Lung function analysis revealed that Rrs and Rn were significantly elevated in the animals treated with OVA compared with control, while challenge with CSE did not alter these parameters. Noticeably, concurrent challenge with OVA and CSE was associated with a significant reduction in Rrs, Rn and G compared with OVA alone (Figure 1B−D).

### 3.2 The effects of CSE on OVA‐induced infiltration of airways inflammatory cells

**FIGURE 2:**
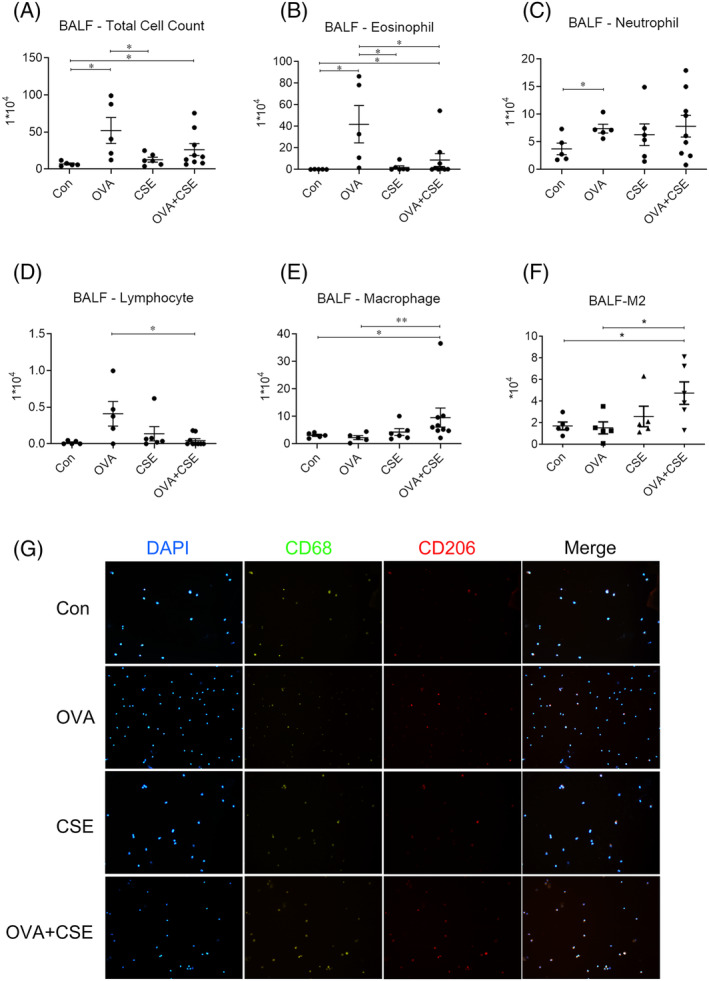
The effects of CSE on OVA‐induced infiltration of inflammatory cells in BALF. (A) Total cell counts in BALF. (B−F) Differential cell counts in BALF. (G) Representative images show nuclei (DAPI, blue) and immunoreactivity for CD68 (green) and CD206 (red) (original magnification ×10). *p < 0.05; **p < 0.01. Data are expressed as the mean ± SEM. BALF, bronchoalveolar lavage fluid; CSE, cigarette smoke extract; M2, alternatively activated macrophages; OVA, ovalbumin.

Cellular analysis of BALF revealed that the total cell counts and number of eosinophils were significantly elevated in the mice challenged with OVA alone or OVA plus serial CSE exposure compared with control, although the latter was significantly reduced in the mice concurrently exposed to OVA and CSE compared with OVA alone (Figure 2A,B). The number of neutrophils in the BALF was significantly increased in the mice challenged with OVA alone compared with control (Figure 2C). The number of macrophages and M2 in the BALF was substantially elevated in the mice concurrently challenged with OVA and CSE compared with OVA alone, which was contrary to the variation of the number of lymphocytes (Figure 2D−G). The mean proportion of M2 in BALF in the mice challenged with OVA alone and OVA plus serial CSE exposure were 6.41% and 38.27%, respectively. Challenge with CSE alone did not significantly alter either total or differential cell counts in the BALF compared with control (Figure 2A−G).

**FIGURE 3:**
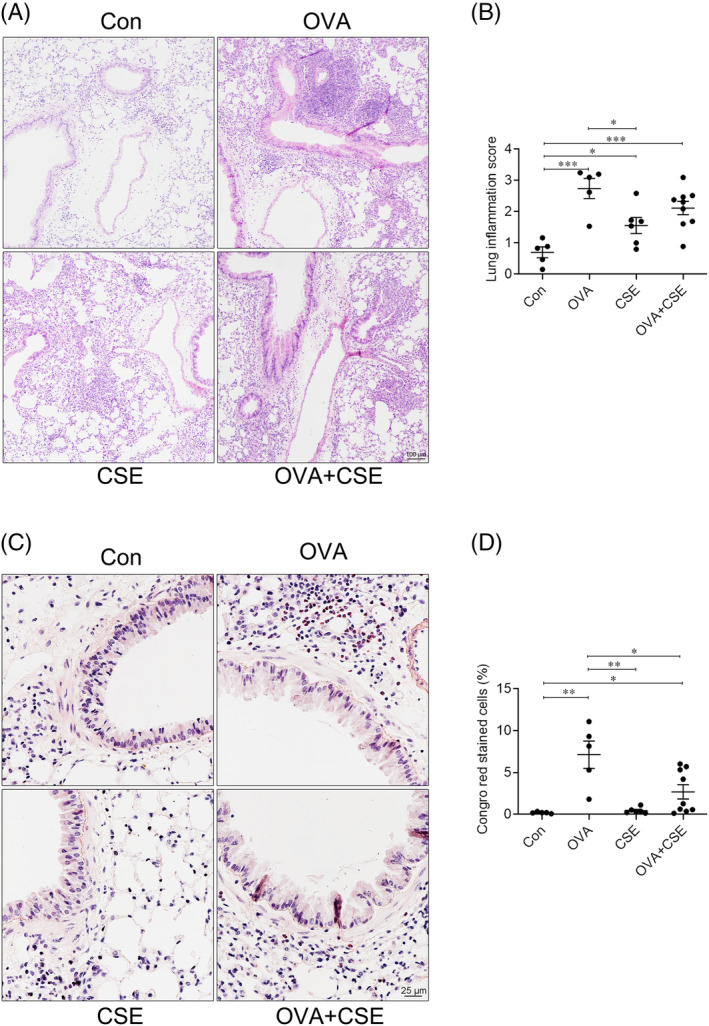
The effects of CSE on OVA‐induced infiltration of total inflammatory cells and eosinophils in lung tissue. (A) Representative photomicrographs of lung paraffin sections stained with HE (original magnification ×10). (B) Inflammatory cellular infiltration score of peripheral airways and vessels. (C) Representative photomicrographs of lung paraffin sections stained with Congo red (original magnification ×40). (D) Semiquantitative analysis of eosinophil infiltration (percentages of total nucleated cells co‐staining with Congo red) in the peripheral airways and vessels. *p < 0.05; **p < 0.01; ***p < 0.001. Data are expressed as the mean ± SEM. CSE, cigarette smoke extract; HE, haematoxylin‐eosin; OVA, ovalbumin.

The infiltration of total inflammatory cells into lung tissue was markedly elevated in the mice challenged with OVA or CSE alone; combined exposure did not significantly alter the effects of each exposure in isolation. Significant eosinophil infiltration, as assessed by Congo red staining, was observed following OVA but not CSE exposure, while concurrent exposure significantly reduced the infiltration observed following exposure to OVA alone (Figure 3).

### 3.3 The effect of CSE on OVA‐induced airways remodelling

**FIGURE 4:**
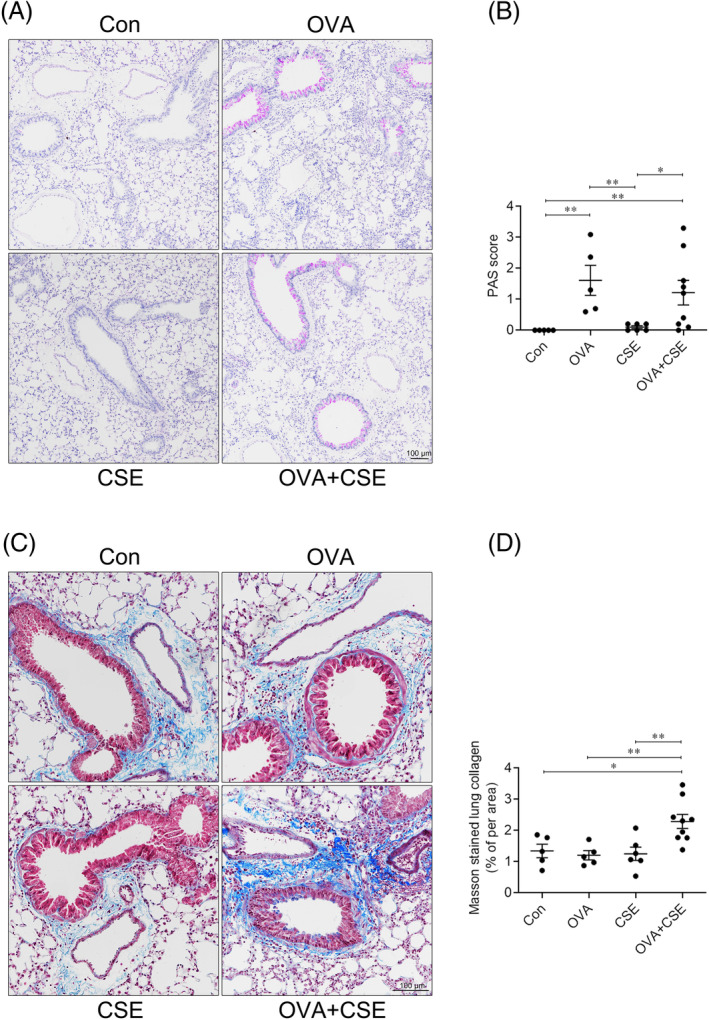
The effect of CSE on OVA‐induced mucous secretion and collagen deposition. (A) Representative photomicrographs of lung paraffin sections stained with PAS (original magnification ×10). (B) Semiquantitative analysis of the PAS scores of the airways. (C) Representative photomicrographs of lung paraffin sections stained with Masson's trichrome (original magnification ×20). (D) Semiquantitative analysis of collagen deposition in the peripheral airways and vessels. *p < 0.05; **p < 0.01. Data are expressed as the mean ± SEM. CSE, cigarette smoke extract; OVA, ovalbumin; PAS, periodic acid Schiff.

Further analysis showed that mucus secretion, as assessed by the PAS score, was significantly increased in the mice challenged with OVA alone or OVA plus serial CSE exposure compared with control, and there was no alteration between treated with OVA alone and OVA plus serial CSE exposure. Additionally, CSE exposure alone did not alter the mucus secretion (Figure 4A,B). Concurrent exposure to OVA and CSE significantly increased collagen deposition in lung of the mice compared with control, OVA or CSE alone, yet there was no alteration in the mice exposed to OVA or CSE alone (Figure 4C,D).

**FIGURE 5:**
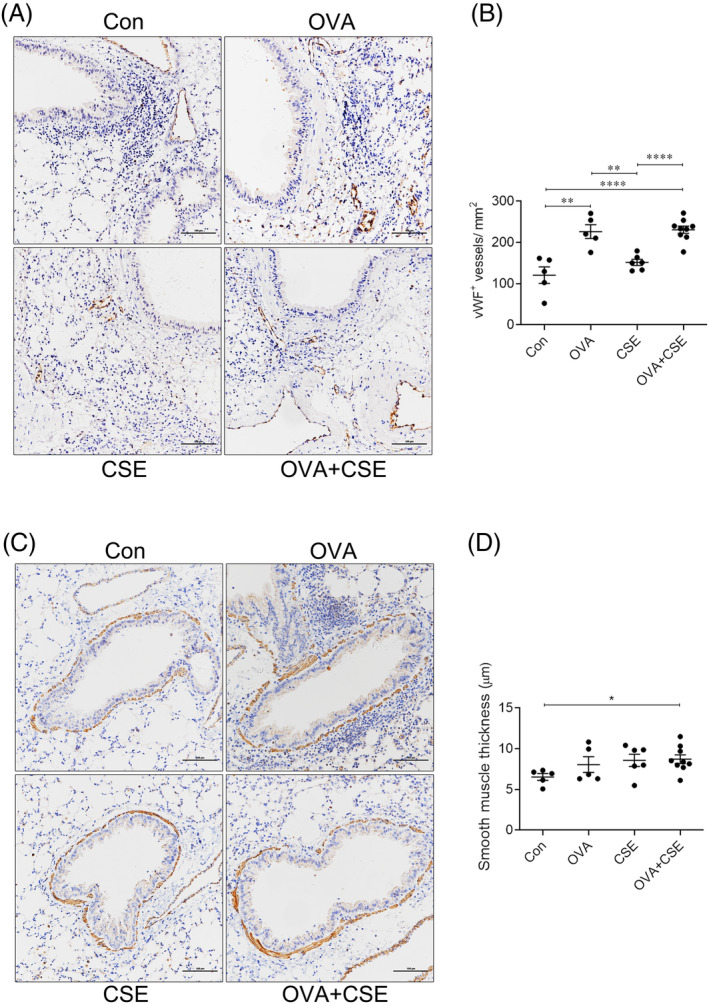
The effect of CSE on OVA‐induced angiogenesis and smooth muscle thickness. (A) Representative photomicrographs of vWF immunoreactivity in lung sections (original magnification ×20). (B) Semiquantitative analysis of the numbers of blood vessels in the lung tissue sections. (C) Representative photomicrographs of immunoreactivity for α‐SMA in the lung tissue sections (original magnification ×20). (D) Semiquantitative analysis of smooth muscle thickness around airways and vessels. *p < 0.05; **p < 0.01; ***p < 0.001. Data are expressed as the mean ± SEM. CSE, cigarette smoke extract; OVA, ovalbumin; vWF, von Willebrand factor; α‐SMA, alpha‐smooth muscle actin.

The number of blood vessels in lung was significantly elevated in the mice challenged with OVA alone or OVA plus serial CSE exposure, but not CSE alone, compared with control, and there was no alteration between treated with OVA alone and OVA plus serial CSE exposure (Figure 5A,B).

Thickness of the smooth muscle layer surrounding airways was significantly elevated in the mice concurrently exposed to OVA and CSE compared with control, while OVA or CSE challenge alone did not significantly alter this index (Figure 5C,D).

### 3.4 The effect of OVA combined with CSE exposure on alveolar structure

**FIGURE 6:**
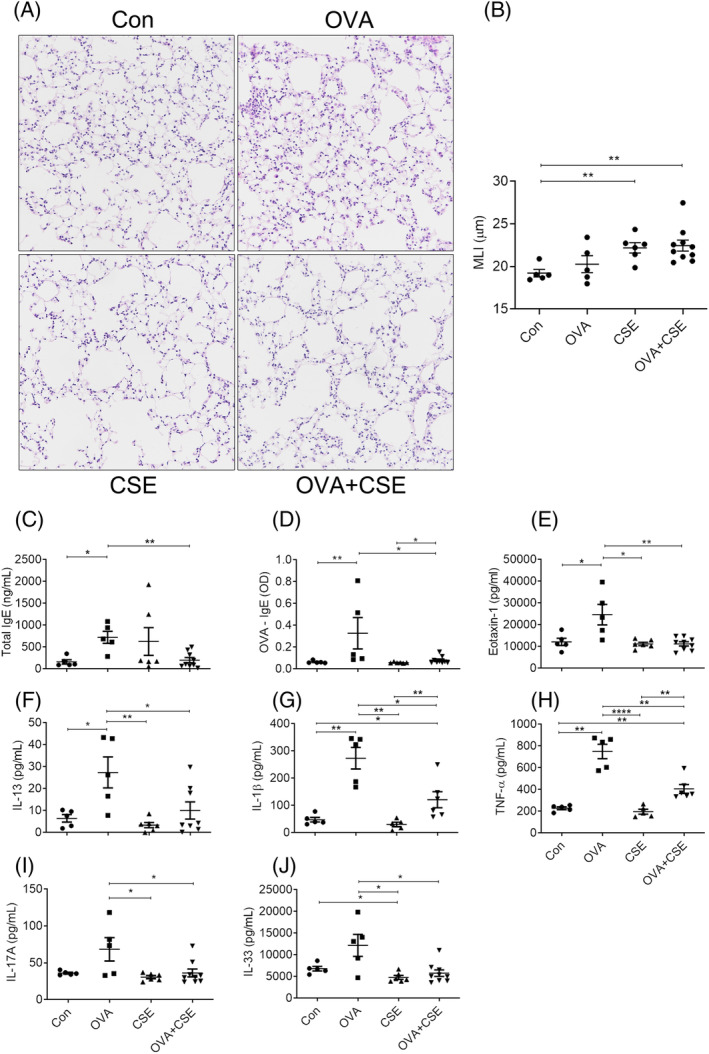
The effect of CSE on OVA‐induced destruction of alveolar structure and expression of IgE and cytokines. (A) Representative photomicrographs of lung paraffin sections stained with HE (original magnification ×10). (B) Semiquantitative analysis of MLI. (C, D) Concentrations of total and OVA‐specific IgE in serum. (E−J) Concentrations of cytokines and chemokines measured in lung homogenates. *p < 0.05, **p < 0.01; ****p < 0.0001. Data are expressed as the mean ± SEM. CSE, cigarette smoke extract; HE, haematoxylin‐eosin; MLI, mean linear intercept; OVA, ovalbumin.

Quantitative analysis of alveolar integrity using MLI revealed that alveolar destruction was significantly increased in the mice exposed to CSE alone or concurrently treated with OVA compared with control, while OVA challenge alone did not alter this index (Figure 6A,B).

### 3.5 The effects of CSE on serum IgE concentrations and expression of cytokines in the lung tissue

As anticipated, sensitisation and exposure of the mice to OVA resulted in a robust and significant elevation of total and OVA‐specific IgE in serum, which were significantly reduced in the mice serially challenged with CSE, whereas CSE exposure alone did not statistically significantly alter these indices compared with control despite clear elevation of the total serum IgE concentrations in some of the experimental animals (Figure 6C,D).

The concentrations of eotaxin‐1, IL‐13, IL‐1β and TNF‐α in lung homogenates of the mice challenged with OVA alone were significantly elevated compared with control, while those were obviously reduced additionally exposed to CSE. Similarly, the expressions of IL‐17A and IL‐33 were also inhibited in the mice concurrent exposed to OVA and CSE compared with OVA alone. The concentrations of IL‐1β and TNF‐α were still significantly higher in the mice concurrently challenged with OVA and CSE than control. In addition, challenge with CSE alone did not alter these parameters compared with control except for the decreased expression of IL‐33 (Figure 6E−J).

## 4 DISCUSSION

It has been estimated that more than one billion people worldwide use tobacco products, mainly through smoking cigarettes, and the prevalence rates of cigarette smokers among asthmatic patients are similar to the general population in many countries or even higher in some countries. Cigarette smoking is typically associated with graver clinical outcomes in some phenotypes of asthma. In addition, mild to moderate asthmatic patients with smoking present an impaired response to inhaled corticosteroid (ICS) therapy.^12^ Although there are many studies associating with smoking and asthma, the entire pathological changes, including AHR, airways inflammation such as inflammatory cells infiltration and the expression of cytokines, as well as airways remodelling like collagen deposition, the thickness of smooth muscle, the number of vessels, mucous secretion and the destruction of alveolar structure, upon the effect of cigarette smoking on asthma remain to be elucidated.

Previous studies have established a clear precedent for the use of the OVA sensitisation and challenge model as a surrogate of the characteristics of human asthma and exposure to soluble extracts of cigarette smoke to explore the effects of cigarette smoke exposure both in vivo and in vitro experiments.^6^, ^8^, ^13^, ^14^, ^15^ Therefore, in the present study, we primarily established a mice surrogate of asthma through OVA sensitisation and challenge and then exposed to CSE to explore the effects of cigarette smoke exposure on the pathophysiological changes of asthma.

It has previously been reported that resistance (Rrs, Rn) is significantly elevated upon methacholine challenge in animal surrogates of asthma, whereas do not change in animal surrogates of emphysema compared with control group.^16^ Of particular interest in our data is inhibitory effect of CSE exposure on AHR responded to methacholine, which is compatible with a clinical research where asthmatic patients with smoking do not appear acute, allergen challenge‐induced AHR to methacholine compared with nonsmoking asthmatic patients.^17^ It has been also shown that low‐dosage carbon monoxide (CO) can abrogate methacholine‐induced AHR, an effect mediated by CO‐induced production of guanosine 3′,5′‐cyclic monophosphothioate (cGMP) mediating airway smooth muscle relaxation.^18^Additionally, nicotine has both anti‐inflammatory and pro‐inflammatory effects, and it has been reported that nictotine reduces mucus production and airways inflmmation in allergic asthma, which in turn influences AHR.^19^ In the present study, we have shown that repeated exposure to CSE, in addition to reducing OVA challenge‐induced AHR, also reduced total cellular infiltration into the airways lumen (BALF) and the bronchial mucosa and interstitial lung tissue of the experimental mice. These findings may be associated with the effects of CO and nicotine.

Eosinophilia can identify asthmatic patients who may benefit more significantly from ICS therapy,^20^ and monoclonal antibodies targeting IgE or the type 2 cytokines (IL‐4, IL‐5 and IL‐13) or alarmins (IL‐33, TSLP) benefit patients with the ‘Type‐2‐high’ phenotype of asthma.^3^, ^21^ Our data showed that airways and tissue eosinophilia, as well as the expression of total IgE, OVA‐specific IgE, IL‐13 and IL‐33 in response to OVA challenge, were inhibited by serial CSE exposure, which may be associated with difficult‐to‐treat asthma with smoking.

Airways remodelling is usually related with more severe phenotypes of asthma, since it contributes to airways obstruction which is less susceptible or resistance to anti‐inflammatory therapy, including ‘biological’ agents.^22^, ^23^, ^24^ The data from the present study are consistent with the hypothesis that CSE exposure is permissive to the development of certain features of remodelling, in particular collagen deposition and smooth muscle hypertrophy in addition to alveolar integrity. Activated macrophages are conventionally subtyped to classically activated macrophages (M1), which are mainly implicated in pro‐inflammatory responses and produce pro‐inflammatory cytokines (IL‐1β, TNF‐α), and alternatively activated macrophage (M2), which are mainly implicated in anti‐inflammatory responses and tissue repair.^25^ The present study showed that the concentrations of IL‐1β and TNF‐α were clearly reduced, while the number of macrophages and M2 were elevated in the mice concurrently challenged with OVA and CSE compared with OVA alone. These implied that M2 might be associated with airway remodelling in the experimental animal concurrent challenge with OVA and CSE.^26^ Additionally, recent advances in single cell RNA sequencing (scRNA‐seq) have facilitated a much more detailed analysis of macrophage function, with the identification of increasing number of pivotal macrophage subsets involved in the occurrence and development of disease.^27^, ^28^ Thus, we suppose that particular subets of M2 type cells may be identified as contributing to the natural history of asthma in patients who smoke, perhaps interacting with fibroblasts, since activated fibroblasts are likely to be the principal producers of collagen and α‐smooth muscle actin,^29^ which were obviously elevated in the experimental mice concurrently exposed to OVA and CSE in the present study. Further studies would be designed to explore the interaction between particular macrophage subsets and fibroblast following the treatment of allergen and cigarette smoke, which may attribute to the changes of airway remodelling in our study.

There are some limitations to our study. Firstly, although CSE is widely used to explore the effects of smoking on health both in vivo and in vitro experiments, it might be argued that direct exposure to smoke via the nasal and respiratory tracts may be more suitable to uncover the full range of its possible effects in asthmatic patients who smoke. Secondly, we have not investigated the potential for conventional anti‐asthma therapies, such as topical bronchodilators and corticosteroids, possibly to modify the interaction between airways hyperresponsiveness, inflammation and smoke exposure in asthma. Finally, as referred to above, the roles of individual immune cells and, ultimately, fibroblasts, airways epithelial and smooth muscle cells and their interaction in asthma with cigarette smoke exposure in the longer term deserve further study in the future.

To conclude, the change of inflammation ‘phenotypes’ and remodelling in asthma following cigarette smoke exposure might contribute to worse symptoms and be refractory to anti‐inflammation therapies for asthmatics, which helps to further understand and explore the mechanism of the pathogenesis of difficult‐to‐treat asthma with smoking.

## AUTHOR CONTRIBUTIONS

**Qiong Huang:** Conceptualization; methodology; investigation; formal analysis; data curation and writing—original draft. **Yan Li:** Methodology; formal analysis and datacuration. **Chenduo Li:** Methodology and investigation. **Xin Zhang:** Methodology and resources. **Xiaonan Du:** Methodology and resources. **Yan Chen:** Supervision andresources. **Chris J. Corrigan:** Conceptualization and writing—review and editing. **Wei Wang:** Conceptualization; supervision; resources and writing—review and editing. **Sun Ying:** Conceptualization; methodology; supervision; resources and writing—review and editing.

## CONFLICT OF INTEREST STATEMENT

The authors report that there are no competing interests to declare.

## ETHICS STATEMENT

All animal experiments were carefully scrutinised and approved by the Institutional Animal Care and Use Committee at Capital Medical University (the reference number: AEEI‐2015‐173).

## Data Availability

The original contributions presented in the study are included in the article; further inquiries can be directed to the corresponding author.
